# Aerococcus urinae Infective Endocarditis

**DOI:** 10.7759/cureus.23947

**Published:** 2022-04-08

**Authors:** Julien Feghaly, Jose Rivas Rios, Malleswari Ravi, Srinivasan Sattiraju, Emil Missov

**Affiliations:** 1 Cardiology, University of Florida College of Medicine – Jacksonville, Jacksonville, USA; 2 Infectious Disease, University of Florida College of Medicine – Jacksonville, Jacksonville, USA

**Keywords:** echocardiogram, echocardiography, rare, endocarditis, infective, aerococcus urinae

## Abstract

*Aerococcus urinae *is a gram-positive organism frequently found in the urinary tract. It is often mistaken for *Streptococcus* and *Enterococcus* based on its appearance. It commonly causes urinary tract infections but has rarely been associated with fatal infective endocarditis and sepsis. We present a case of *Aerococcus urinae* infective endocarditis and discuss echocardiographic imaging findings and management approach.

## Introduction

*Aerococcus urinae *(*A. urinae*)* *is a gram-positive catalase-negative alpha-hemolytic coccus that grows in clusters and is commonly found in the urinary tract [1]. It is characterization can often be challenging, as *A. urinae* may resemble an alpha-hemolytic streptococcus or enterococci at 24-48 hours. Once thought of as a rare human pathogen, *A. urinae* is now increasingly identified as a human pathogen with the advancements in species determination methodologies, such as matrix-assisted laser desorption ionization time-of-flight mass spectrometry (MALDI-TOF MS) [2,3].

## Case presentation

A 79-year-old male with a history of atrial fibrillation with WATCHMAN^TM^ (Boston Scientific, Marlborough, Massachusetts) implantation, complete heart block with a dual-chamber pacemaker in situ, ischemic stroke, end-stage renal disease on hemodialysis, hypertension, and diabetes mellitus type 2, presented with shortness of breath, left hip pain, non-bloody diarrhea, and a productive cough. He denied chest pain, fever, chills, lower extremity edema, orthopnea, or abdominal pain. He had a former history of tobacco use but no alcohol or illicit substance abuse disorders.

**Figure 1:**
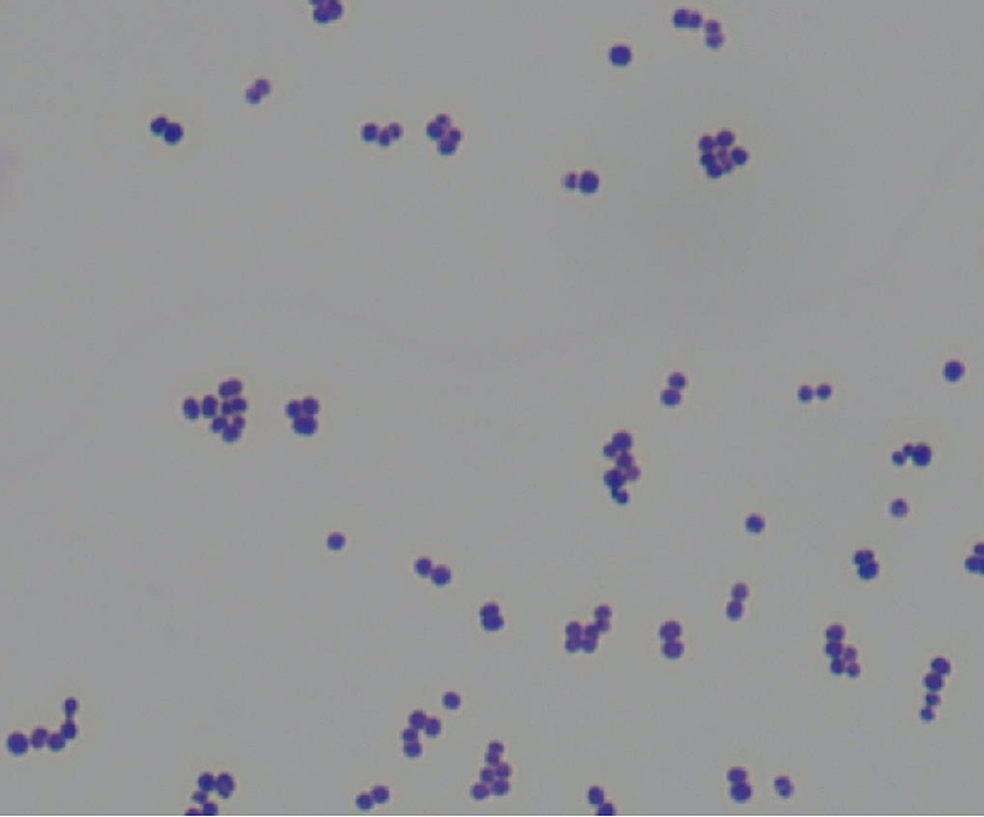
Gram stain of Aerococcus urinae *Aerococcus* species appear as gram-positive cocci in clusters.

On presentation, he was hemodynamically stable, afebrile, and had no oxygen requirements. A chest X-ray revealed bilateral interstitial opacities suggesting cardiopulmonary edema. CT of the abdomen/pelvis revealed mild cardiomegaly, mitral valve calcifications, and findings suggestive of congestive heart failure, including small bilateral pleural effusions, trace ascites, mesenteric stranding, and mild anasarca. Laboratory tests were notable for elevated white blood cell count with 96% neutrophils and normal lactic acid level. Vancomycin, cefepime, and metronidazole were initiated empirically. Blood cultures collected on admission grew *A. urinae* (Figure 1).

**Figure 2:**
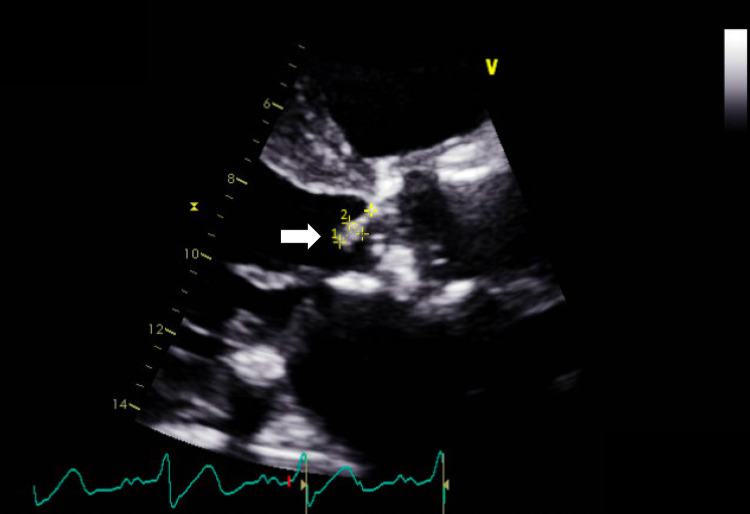
Transthoracic echocardiogram parasternal long axis aortic valve focused view An approximately 1.2 x 0.4 centimeter echogenic density (arrow) is attached to the aortic valve towards the left ventricular outflow tract.

Transthoracic echocardiogram revealed an ejection fraction of 40-45%, mild aortic stenosis (peak gradient 2.7 m/s, mean gradient 17 mmHg), a 1.2 x 0.4 centimeter density on the aortic valve suggestive of vegetation (Figure 2, Video 1), moderate aortic regurgitation, and moderate mitral regurgitation.

**Video 1:** Transthoracic echocardiogram parasternal long axis aortic valve focused view An approximately 1.2 x 0.4 centimeter echogenic density is attached to the aortic valve towards the left ventricular outflow tract.

A transesophageal echocardiogram revealed a large 1.5 x 0.5 cm mobile vegetation in the left ventricular outflow tract (Video 2) that crossed the aortic valve plane and was associated with severe aortic regurgitation (Video 3). Pacemaker leads had no convincing evidence for vegetations. Antibiogram revealed *A. urinae* susceptible to ceftriaxone, penicillin, and vancomycin. The antibiotic regimen was adjusted to vancomycin and gentamicin. The patient's shortness of breath gradually improved, and the left hip pain was attributed to osteoarthritis and self-resolved during the admission. Electrophysiology and cardiothoracic surgery services were consulted for pacemaker and lead extraction and aortic valve surgery. However, the patient was deemed not to be a surgical candidate due to comorbidities. Following a multidisciplinary discussion, it was decided to pursue medical management with vancomycin monotherapy for six weeks from the first negative blood culture date and to discharge with out-patient cardiology follow-up.

**Video 2:** Transesophageal echocardiogram mid-esophageal long axis view Large 1.5 x 0.5 centimeter mobile vegetation in the left ventricular outflow tract that crosses the aortic valve plane.

**Video 3:** Transesophageal echocardiogram mid-esophageal short-axis view Aortic valve is structurally abnormal and associated with severe aortic regurgitation.

Unfortunately, he was subsequently readmitted for encephalopathy and worsening respiratory status after having completed the treatment course with vancomycin. A repeat echocardiogram showed persistence of vegetations and severe aortic regurgitation. Given his worsening clinical status and following goals of care discussions, the decision was made to pursue comfort care.

## Discussion

*A. urinae *previously was considered an organism with low pathogenicity believed to rarely cause urinary tract infections in elderly males with a history of urinary illness or following urological interventions [2]. However, *A. urinae *and *Aerococcus sanguinicola *are now increasingly associated with infective endocarditis and sepsis [4]. Its potential virulence is attributed to platelet aggregation and biofilm formation, increasing the likelihood of infection in patients with implanted cardiac devices and underlying valvular disease [5].

Prior case reports on *A. urinae *or aerococcus-like organism endocarditis have been published in the literature, yet aerococcal bacteremia remains rare [6]. The presence of *A. urinae* infective endocarditis carries a poor prognosis with a mortality rate of 50%. This in turn may be related to a delay in the identification and initiation of appropriate antibiotic regimens or due to patient-specific characteristics such as age or underlying cardiac disease [2,7]. This data, however, may be skewed by published bias [6].

Despite the most common source of aerococcal infection, bacteremia being in the urinary tract, it may often go unidentified despite symptoms of urinary tract infections [6]. This may be a result of urine cultures not being incubated in a carbon dioxide-containing environment allowing for aerococci growth [2,3,6]. Other presentations may include genital soft-tissue infections, postpartum infections, vertebral osteomyelitis, joint infections, peritonitis in patients, and postoperative infections [8-11].

An optimal antibiotic approach and duration have yet to be defined. A treatment regimen using multiple antibiotics (penicillin G and aminoglycosides) is often an effective option. Another suitable alternative is vancomycin or ceftriaxone. Antibiotics choice should then be further guided by variable species susceptibility patterns [12]. Management should additionally include infection source identification, followed by source control or device extraction where appropriate and feasible.

## Conclusions

This case highlights a rare case of *A. urinae* infective endocarditis, which likely carries a high mortality, especially in the elderly population. Prompt diagnosis and initiation of antibiotic therapy is believed to be imperative for treatment and improving patient outcomes. Transthoracic and transesophageal echocardiography aid in the diagnosis and evaluation of structural and valvular abnormalities. Once the diagnosis is confirmed, source control needs to be achieved when feasible and multidisciplinary assessment is necessary for determining optimal treatment.
